# Supplementary material for: Are carbon emissions trading and green financial instruments synergistic? -Comprehensive quantitative research based on content analysis
Source: PLoS One. 2024 Mar 7;19(3):e0298601. doi: 10.1371/journal.pone.0298601 (PMC10919601; doi:10.1371/journal.pone.0298601)
Supplement: S1 Data — (ZIP) [file pone.0298601.s001.zip › Data/地级市Z_F工作报告.zip!/║■▒▒/╬Σ║║╩╨/└┤╫╘╣1⁄2╓┌║┼í╛┬φ┐╦╩2╛▌═°í┐_2019.docx]

各位代表：

现在，我代表市人民政府，向大会报告工作，请予审议，并请市政协各位委员和列席人员提出意见。

一、2018年工作回顾

刚刚过去的一年，是我国改革开放40周年，是武汉发展史上具有里程碑意义的一年。习近平总书记再次视察武汉，为新时代武汉高质量发展定向导航、把脉施治。武汉发展站上新起点，迈入新境界。

一年来，全市上下深入学习贯彻党的十九大精神和习近平总书记视察湖北重要讲话精神，牢记“四个切实”殷殷嘱托，在省委省政府和市委的坚强领导下，高扬干事激情，迸发奋进力量，紧紧围绕建设现代化、国际化、生态化大武汉和国家中心城市，坚持一张蓝图干到底，对标先进、拼搏赶超，有效应对各种困难和挑战，努力做到“作风扎实、工作落实、结果真实、心里踏实”，圆满完成了市十四届人大二次会议确定的目标任务，高质量发展取得新成效。

——主要经济指标增速保持全国同类城市前列。以第一力度抓第一要务，主要经济指标实现全面增长，预计地区生产总值增长8%左右，全社会固定资产投资增长10.5%左右，社会消费品零售总额增长10%以上，地方一般公共预算收入同口径增长11%。

——发展新动能加速集聚。强化创新战略支撑，加快建设存储器、航天产业、网络安全人才与创新、新能源和智能网联汽车四个国家新基地，着力打造光电子信息、汽车及零部件、生物医药及医疗器械三大世界级产业集群，推动产业优化升级，“三新”经济增加值占经济总量比重超过27%，科技进步贡献率超过60%。

——全民迎军运热潮涌动。坚持细致精致极致卓越，聚焦“五边五化”，全域推进综合环境整治提升，深入开展“与军运同行”系列活动，环境面貌加速蝶变，大江大湖大武汉的城市魅力进一步彰显，市民热情持续高涨，“迎接军运会、打造新环境、当好东道主”成为全民行动。

——一批痛点难点问题加快破解。坚持以人民为中心，全力打好三大攻坚战，用改革的办法积极破解发展中的难题，大力优化营商环境，“放管服”改革走在全国前列；大力推进“一城一港一主体”，阳逻国际港实现统一运营；大力整治城市黑臭水体，水质提升攻坚取得积极进展；大力建设停车场、充电桩，市民反映强烈的停车难问题正逐步缓解。十件实事全面完成。

——城市影响力显著提升。50多项国家重大战略和改革试点任务叠加聚焦，武汉在国家区域协调发展战略中，被赋予引领长江中游城市群发展新使命。外国元首、政府首脑、国际政要和商业巨头频频来访，中国国际友城大会、中外企业家峰会、大河对话等重大国际活动接连举办，武汉正成为海内外关注的热点城市。

过去的一年，面对外部环境明显变化、经济下行压力加大、城市竞争日益激烈，全市上下勠力同心、迎难而上，表现出强烈的大局意识、进取精神和拼搏劲头，交出了一份逆势前行、强劲发展的年度答卷!

一年来，我们主要做了以下工作：

一是坚持高位求进，综合经济实力加快跃升。实施万千百工程，着力提升产业能级，光电子信息、汽车及零部件、生物医药及医疗器械三大产业，主营业务收入总量突破5000亿元；金融、商贸、物流、建筑等行业增加值均过千亿元；新兴产业领域亮点纷呈，电子商务交易额8200亿元，增长25%，软件和信息技术服务业营业收入突破2100亿元，增长17%以上；旅游、会展、设计等产业加快发展。做大做强市场主体，在汉投资世界500强企业达到266户，中国信科、国华人寿、长江生态环保集团等企业总部落户武汉，6户企业进入中国企业500强，10户企业进入中国民营企业500强；长飞光纤、锐科激光、明德生物等9户企业成功上市，总数达77户，居中部城市首位，市场主体达到118万户。深入实施招商引资“一号工程”，举办粤港澳大湾区、环渤海等13场大型招商活动，签约金额2.01万亿元，实际到位资金8600亿元，实际利用外资增长13.3%，签约投资亿元以上项目559个、50亿元以上工业项目23个、30亿元以上服务业项目62个。大力开展“项目落地建设竞赛年”活动，组织集中开工活动9次，开工项目总投资8889亿元，长江存储、东风新能源汽车产业园、翰宇药业等5个投资50亿元以上工业项目建成投产，武烟易地技改工程完成。强化区级经济支撑，国家级开发区产业集聚能力不断增强，大项目扎堆落户；中心城区现代服务业创新升级，涌现出50余栋纳税亿元以上商务楼宇；新城区现代产业园加快建设，工业占经济总量比重稳步提升。大力实施乡村振兴战略，“三乡工程”拓面提质，吸引社会资金156.9亿元，乡村休闲游综合收入140亿元。武汉开发区、东湖高新区、江汉区、武昌区、江岸区、汉阳区、洪山区经济总量过千亿元。推动军民融合深度发展，建立“两库一清单”，积极解决军工企业搬迁、周边环境整治等问题。第四次全国经济普查全面推进。武汉促进工业稳增长和转型升级、打造新兴产业集群等做法受到国务院通报表扬。

二是坚持创新引领，内生增长动力加快壮大。深入推进全面创新改革试验，全社会研发经费投入（R&D）增长10%以上，入选国家知识产权运营服务体系建设城市，发明专利申请量28500件、授权量9717件，每万人发明专利拥有量33.1件，居全国同类城市前列，成功突破100G硅光收发芯片、自主架构64层三维闪存芯片、氢油物流车等一批前沿技术。加快高端创新平台建设，国家制造业创新中心增至2个，成为比肩北京、上海的“双中心”城市；新增诺贝尔奖工作站2个、院士专家工作站28个、知名企业研发机构47个、省级以上创新平台41个、省级众创孵化机构51个。新引进外籍外地院士45名、高层次人才281名；留汉创业就业大学毕业生40.6万名，创历史新高，人口净流入量居全国城市前列。持续推动科技成果转化对接，举办专场活动216场，1072项重大科技成果就地转化，签约总金额371.4亿元；技术合同成交额突破700亿元。科技金融保持活跃，创投资本总额达到1337.9亿元，增长9.1%。新产业新业态新模式蓬勃发展，净增高新技术企业700户以上，新增科技“小巨人”企业1237户、瞪羚企业500户；独角兽企业达到5户，居全国第5位；高新技术产业产值突破1万亿元。

三是坚持改革开放，城市发展活力加快释放。深化供给侧结构性改革，清理处置僵尸、空壳类亏损国有企业31户，为企业减税降负279亿元。成功创建全国质量强市示范城市，中铁大桥局荣获中国质量最高奖，实现湖北“零”的突破。聚焦营商环境，深化“放管服”改革，市级“网上办”事项达到73.1%，大幅压减行政审批时限，企业开办、工程建设项目审批时间实现“双减半”，商事制度改革、政务服务改革经验受到国务院通报表扬。坚持“两个毫不动摇”，出台民营经济高质量发展政策措施，发放小微企业融资应急资金33.3亿元，帮助企业获得银行续贷融资38.5亿元，为5000户以上中小民营企业提供生产、财务、市场等免费培训，民间投资占全市一半以上。国资国企改革稳步推进，混合所有制改革取得新进展，全面完成市级部门所属企业脱钩任务，首次实现国资监管全覆盖。实施控房价、稳市场和刚需家庭优先购房措施，房地产市场保持平稳健康发展。建立金融风险监测预警防范化解体系，坚决遏制非法集资、非法金融等活动，区域性金融风险总体可控。大力推进自贸区武汉片区建设，全面落实自贸区负面清单，新注册企业10547户，新引进跨国公司研发机构11户，“先出区、后报关”等4项创新举措在全国复制推广。经开综保区获批设立，武汉成为第2个拥有3个综保区的副省级城市。国际经济合作迈出新步伐，对外经济合作营业额增长19%，获批建设国家跨境电商综合试验区、国家深化服务贸易创新发展试点城市。进一步拓展国际大通道，新开通至伦敦等国际及地区航线5条，天河国际机场实现7×24小时常态化通关；中欧（武汉）国际班列延伸至34个国家、76个城市，是全国唯一进口列数超过出口列数的班列。加强对外交流合作，成功举办国际性大型活动20余场次，新增曼谷等5个国际友好城市和友好交流城市，总数达109个。侯卫东官场笔记

四是坚持强基提质，城市功能体系加快完善。强化规划先导作用，城市总体规划、长江新城总体规划编制完成，快速路网、轨道交通等专项规划加快修编，东湖绿道获国际规划卓越奖。完善综合交通体系，长江中游航运中心建设加快推进，江海直达千箱级集装箱示范船成功首航，阳逻国际港集装箱吞吐量达到145.7万标箱，增长14.9%；地铁7号线一期、11号线东段一期、纸坊线开通试运营，通车总里程达305公里，轨道交通第四期建设规划获国家批复；硚孝高速公路二期开工建设，长江公铁隧道、月湖桥拓宽改造等工程竣工通车，四环线南段基本建成，江北快速路全线贯通，武九铁路北环线启动搬迁；建成微循环道路122条，新增停车位10万个，充电桩累计达10万个。深化“智慧交通”建设，武汉交通拥堵状况连续五年好转，获批国家“公交都市”建设示范城市。实施城管精细化提标行动，户外广告减量提质，建筑立面、城市天际线、“三站一场”、背街小巷等专项整治深入开展，“三旧”改造扎实推进。“厕所革命”成效明显，新改扩建城镇公共厕所345座、旅游厕所104座，改造农村户厕9.6万户。千子山、长山口等垃圾处理项目加快推进，生活垃圾分类试点年度任务全面完成。着力提高防洪排涝能力，长江汉江干堤、中小河流堤防进一步巩固，全市新增抽排能力404.5立方米/秒，开工建设海绵城市40平方公里。改造235处老旧小区二次供水设施。扎实做好中央、省生态环保督察及有关环保专项督查反馈意见整改工作。纵深推进污染防治攻坚战，打好长江大保护十大标志性战役，开展“五大行动”，实施南湖、汤逊湖等重点水体水质提升攻坚，新增城镇污水处理能力48.5万吨/日，增长16.2%，PM10、PM2.5平均浓度分别下降14.1%、5.8%，土壤污染治理修复有力推进。开展增绿提质行动，建设各类公园42个，新增绿地758万平方米，新建绿道116公里，提档升级道路绿化景观550公里，东湖绿道三期全面建成，精准灭荒任务提前完成。

五是坚持民生优先，市民生活水平加快提高。持续加大民生投入，公共财政用于民生领域支出1402.7亿元，增长15%。突出产业扶贫，全力打好精准脱贫攻坚战，全市实现减贫21597人，87个贫困村出列。积极推进就业社保拓面提质，新增就业21.9万人，社会保险净增参保98万人次，退休人员基本养老金月人均上调160元，城乡低保综合提标11.4%。成立退役军人事务局。深入推进养老服务业综合改革，建成老年宜居社区600个，新改扩建社区老年人服务中心（站）96家，新建农村老年人互助照料活动中心（服务点）302个。加大住房保障力度，实施棚户区改造5.2万户，建成保障房3.8万套，筹集长租房源11.2万套，将外来务工人员纳入公租房保障范围，为2460个老旧小区提供物业服务，推进老旧小区加装电梯。深化教育综合改革，新改扩建公益普惠性幼儿园54所、中小学校31所，新增学位近6万个。深化医药卫生体制改革，医疗费用增幅和药占比持续下降，全市二级以上公立综合医院全部开设儿科独立病区，光谷同济儿童医院、武汉开发区儿童医院开工建设，为15万名适龄妇女免费开展“两癌”筛查。完成残疾人家庭无障碍改造1万户。加大优质公共文化服务供给，开展文化惠民活动1000余场，发放旅游惠民券100万张，武汉自然博物馆建成开放，盘龙城遗址博物院建成试运行，武汉马拉松连续实现“零差评”，武汉卓尔足球队冲超成功。深化平安武汉建设，扎实推进扫黑除恶专项斗争，刑事警情下降22.4%。深入开展市区领导干部每月下基层大接访，进一步发挥“网上群众工作部”平台作用，加快化解信访积案。安全生产形势总体平稳，实现“一无一降”目标。

在做好各项工作的同时，我们狠抓政府作风建设，注重保持工作连续性、创新性，强化“执行力是政府第一能力”，加强督查督办，大力营造雷厉风行抓落实的新气象，严格落实全面从严治党主体责任，深入推进政府系统党风廉政建设，积极开展省委巡视反馈问题整改，深化法治政府建设，自觉接受各方面监督，办理市人大代表建议380件、政协提案700件。

国家安全、国防动员、消防救援、民防、仲裁、保密、气象工作扎实推进，参事、文史、档案、民族、宗教、对台、侨务、地方志工作不断加强，妇女、儿童、老龄、残疾人事业取得新进步。

各位代表，为者常成，行者常至。过去一年取得的成绩，是习近平新时代中国特色社会主义思想指引的结果，是以习近平同志为核心的党中央英明领导的结果，是省委省政府坚强领导的结果，是市委团结带领全市人民克难攻坚、拼搏赶超的结果，凝聚了全市方方面面的智慧和汗水。在此，我代表市人民政府，向全市人民，向给予政府工作大力支持的人大代表、政协委员，向各民主党派、工商联、人民团体和社会各界人士，向中央在汉单位和各类驻汉机构，向驻汉人民解放军和武警官兵，向参与城市建设的劳动者，向所有关心支持武汉发展的港澳台同胞、海外侨胞和国际友人，表示崇高的敬意和衷心的感谢！

我们也清醒地认识到，对照高质量发展要求，对标先进城市，还有不少差距和短板，主要表现为：质量、效率、动力变革有待进一步深化，创新尚未真正成为引领发展的第一动力，新动能对经济的支撑作用还不稳固；市场化改革还需加大力度，民营经济、开放型经济发展还不充分，国际化水平还不高；环境污染问题还没有得到根本性解决，河湖水质提升任务仍然较重，城市精细化管理还需加强；民生保障还存在短板，教育、医疗、养老等领域优质服务供给还不充足；营商环境还需进一步优化，少数政府工作人员作风不实，还存在不担当、不作为的现象。面对这些问题，我们不回避、不畏难，一步一步推进，一件一件解决，努力让人民群众感受到实实在在的变化。

二、2019年重点工作任务

今年是新中国成立70周年，第七届世界军人运动会将在武汉举办，做好今年工作，对于决胜全面建成小康社会、建设“三化”大武汉和国家中心城市至关重要。

当前，武汉正处于高质量发展关键阶段。我们要牢牢把握重要战略机遇期，积极应对各种风险挑战，保持高质量发展良好势头。习近平总书记两次视察武汉，为武汉发展指明了方向，全市上下形成了感恩奋进、拼搏赶超的强大气场。国家区域协调发展战略赋予武汉新使命，进一步强化了武汉“国家战略要津”的地位。经济总量在万亿台阶上持续攀升，各种有利因素不断增多，为武汉发展奠定了坚实基础。我们要坚定信心、把握主动，加快提升城市能级和核心竞争力，努力在全国全省改革发展大局中体现更大担当、更大作为！

今年政府工作的总体要求是：以习近平新时代中国特色社会主义思想为指导，全面贯彻党的十九大和十九届二中、三中全会精神，牢记“四个切实”殷殷嘱托，统筹推进“五位一体”总体布局，协调推进“四个全面”战略布局，按照全省“一芯驱动、两带支撑、三区协同”的高质量发展区域和产业战略布局要求，坚持稳中求进工作总基调，以供给侧结构性改革为主线，以改革开放创新为动力，继续打好三大攻坚战，统筹推进稳增长、促改革、调结构、惠民生、防风险工作，扎实做好“六稳”工作，着力增强人民群众获得感、幸福感、安全感，努力保持经济持续健康发展和社会大局稳定，争做“五个排头兵”。

今年的主要预期目标是：地区生产总值增长8%左右，地方一般公共预算收入增长8%左右，全社会固定资产投资增长11%左右，社会消费品零售总额增长10.5%左右，城乡居民人均可支配收入增长和经济增长基本同步，全面完成省下达的节能减排任务。

重点抓好以下八个方面工作：

（一）全力打好三大攻坚战，夯实全面建成小康社会基础

打好防范化解重大风险攻坚战。注重关口前移，加强地方法人金融机构管理，完善风险监测预警机制，妥善处置非法集资、互联网金融、重点企业债务等金融风险，守住不发生区域性金融风险的底线。加强政府债务预算管理和限额管理，规范政府举债融资行为，稳妥化解存量政府债务，严格控制新增隐性债务，加快推进融资平台公司转型。坚决防范社会风险，扎实开展矛盾纠纷大排查、隐患大整改、治安大整治，防止各类矛盾叠加集聚，防范化解社会稳定、公共安全、网络安全等风险。

打好精准脱贫攻坚战。深入实施脱贫攻坚八大工程，推进乡村振兴与脱贫攻坚统筹融合。注重扶贫与扶志相结合，增强贫困户内生动力，加强对贫困边缘人口政策支持，建立稳定脱贫和防止返贫长效机制，确保已脱贫人口和村不返贫。

打好污染防治攻坚战。建立健全环境污染防治长效机制。打好碧水保卫战，开展“清源”“清管”“清流”行动，推动南湖、汤逊湖、东湖、北湖等水体质量持续改善，全面消除城市建成区黑臭水体，严格管控农业面源污染。打好蓝天保卫战，聚焦工业生产、移动源排放、扬尘、燃煤四大污染源，强化综合治理系列措施，进一步提高空气质量优良率，推进PM10、PM2.5平均浓度持续下降。打好净土保卫战，全面整治工业废物堆存场所，加快推进土壤污染治理修复。扎实做好中央、省生态环保督察及有关环保专项督查反馈意见整改工作。

（二）加快产业优化升级，推动经济高质量发展

推动制造业高质量发展。紧盯关键领域和薄弱环节，开展补链、强链、延链行动，大力推进光电子信息、汽车及零部件、生物医药及医疗器械三大世界级产业集群建设，规模以上工业增加值增长7.8%左右。确保东风本田三厂、京东方10.5代线、华星光电T4等5个投资50亿元以上项目投产。加快推进长江存储二号厂房、天马G6项目二期、东风乘用车扩建等18个投资50亿元以上项目建设，工业投资增长13%以上。加快先进制造业和现代服务业深度融合，开展工业新技改行动，加快国家工业互联网顶级节点建设和创新应用，滚动实施100个企业技改示范项目，加大宝武武钢集团等重化工企业技改支持力度，推进规模以上工业企业技改项目全覆盖，提升装备制造、钢铁、石化、日用轻工、建材等优势产业竞争力。全面开展质量提升行动，积极创建国家标准化改革创新先行区，着力打造一批新名企、新名家、新名品。

推动现代服务业创新升级。实施服务业高质量发展八大行动，加快建设“一都一枢纽、两城四中心”，服务业增加值增长8.5%以上。支持中心城区建设现代服务业集聚区，大力发展楼宇经济，力争新增5栋纳税亿元以上商务楼宇。大力发展总部经济，支持东风汽车、中国信科、宝武武钢集团、葛洲坝集团、中诚信、小米武汉等各类总部企业跨越发展，支持更多行业龙头企业和知名企业在汉举办全球供应商大会，大力引进企业总部或“第二总部”，加快发展数字经济、共享经济。深入实施新消费引领计划，鼓励发展生鲜电商、智慧零售等新业态，电子商务交易额增长20%以上,培育引进知名品牌旗舰店、体验店50个。推进全市商圈差异化发展，支持各区打造商业地标，推动汉正街高品质发展，高标准、高品位建设江汉路步行街，带动中山大道及周边商业功能品质提升，鼓励社会资本复兴“老字号”。推动市属商业集团转型发展，提升本土商贸企业竞争力。

积极扩大有效投资。市级重大项目计划投资2200亿元以上。积极支持民间投资健康发展，有序推进政府和社会资本合作项目，不断激发民间有效投资活力。深化招商引资“一号工程”，实际到位资金8600亿元以上，实际利用外资增长12%。推动与世界500强企业、中国企业500强等知名企业深度对接合作。聚焦全市主导产业和战略性新兴产业，大力引进关键环节、核心企业和上下游配套项目,打造完整产业链。

支持民营经济健康发展。着力破解民营企业融资难，逐步形成总规模100亿元制造业发展基金、总额100亿元上市公司纾困基金，筹措100亿元用于支持融资担保体系建设。开展民营企业壮大工程，扩大“四上”企业总量规模，加快培育中国民营企业500强。构建亲清新型政商关系，搭建政商交往、政企沟通制度化平台，深入开展领导干部联系服务企业等活动，切实为民营企业排忧解难。

加快建设区域金融中心。深入推进武汉科技金融改革创新试验区建设，申报国家级保险型社会先行示范区。增强金融服务实体经济能力，持续推进上市公司倍增三年行动计划，积极支持企业在科创板上市，新增境内外上市企业8户以上、上市后备“金种子”企业100户，直接融资规模达到2000亿元。开展常态化银企对接活动，实施金融支持小微企业“万企千亿”行动计划，确保实现“两增两控”目标。做大做强法人金融机构，新增金融机构10户以上。

（三）着力完善创新体系，争创综合性国家产业创新中心

推进“一芯驱动、两带支撑、三区协同”战略布局落地落实。全面提速存储器、航天产业、网络安全人才与创新、新能源和智能网联汽车四个国家新基地建设，大力发展以集成电路为代表的高新技术产业、战略性新兴产业和高端成长型产业，培育“国之重器”的“芯”产业集群。集聚创新要素资源，推动形成绿色发展方式和生活方式，加快构建现代产业体系，争当全省高质量发展第一动力源，在长江绿色经济和创新驱动发展带、汉随襄十制造业高质量发展带中发挥更大作用。

提高自主创新能力。推动高校院所与龙头企业资源共享对接，积极筹备建设国家实验室，提升脉冲强磁场、生物安全与技术等大科学设施功能，提升光电国家研究中心、信息光电子创新中心、数字化设计与制造创新中心、先进存储产业创新中心等平台自主创新能力，谋划建设一批重大科技基础设施。加强生命科学、人工智能、量子科学、未来网络等领域战略布局，力争突破一批前沿领域核心技术。

建设科技成果转化高地。支持武大、华科大等在汉高校“双一流”建设，推进市属高校内涵特色发展。深化高校院所科技成果转化对接工程，力争签约转化项目800个以上。提升武汉技术交易市场功能，建成全国高校技术转移华中中心。推动工研院创新发展，支持建设一批高水平工研院。大力发展院士经济，加强与国内外院士对接合作，新建院士专家工作站20个，力争落地院士合作项目50个以上。实施新技术新产品应用示范工程，建立“首购首用”风险补偿机制，促进装备首台套、新材料首批次、软件首版次产品的应用推广。

实施高新技术企业倍增计划。新增高新技术企业800户，3年内突破6000户。培育科技型高成长企业1500户。大力发展“专精特新”企业，加快培育“隐形冠军”“单项冠军”。鼓励企业加大研发投入，新增省级以上创新平台50家。深入开展知识产权强市创建，发明专利申请量、授权量均增长13%。

推动军民融合深度发展。促进创新资源共享，加强军地联合攻关，提高军民协同创新能力。推进“军转民”和“民参军”双向转化，加快推动实施174个军民融合项目，大力支持船舶与海工装备、智能装备、航空航天、新一代信息技术、医疗卫生等军民融合优势产业发展，积极争创国家军民融合创新示范区。

打造“双创”升级版。实施全域推进国家自主创新示范区建设行动，规划建设光谷科技创新大走廊，加快建设环大学创新经济带，新增省级以上众创孵化机构30个。深入开展招才引智，更大力度吸引大学生留汉创业就业，更大力度引进海内外高层次人才和团队，新增留汉大学毕业生25万名，引进战略科技人才10名、产业领军人才200名。大力实施“武汉工匠”培育计划。促进科技资源共建共享，推动一批大型科技仪器向社会用户开放。

（四）全面深化改革，打造内陆开放新高地

深化重要领域改革。深入推进供给侧结构性改革，在“巩固、增强、提升、畅通”上下功夫。完成全面创新改革试验任务，形成一批可复制可推广的经验。实打实推进国资国企改革，深化市属国企混合所有制改革，做强做优做大国有资本。推进稳地价、稳房价、稳预期“一城一策”试点，培育发展住房租赁市场。推动住房公积金制度改革。健全预算绩效管理体系。推进供销合作社综合改革。

打造一流营商环境。开展营商环境重点领域提升行动。全面实施市场准入负面清单制度，营造公开公平公正的营商环境。深化“放管服”改革，推进马上办、网上办、就近办、一次办，实现企业开办时间压减至3个工作日以内、工业项目施工许可压减至50个工作日以内、工程建设项目审批压减至90个工作日以内，强化事中事后监管，完善“双随机一公开”监管机制。开展降本增效专项行动，以更大力度降低企业税费、融资、物流、用工等成本，实质性减轻企业负担。营造法治化制度环境，维护企业合法权益，加强产权保护，创建国家社会信用体系建设示范城市。强化以评促建，全面开展营商环境考核评价。

构建全方位对外开放新格局。发挥自贸区武汉片区示范引领作用，突出“双自联动”，加大金融创新力度，强化产业支撑，培育引进国际化市场主体，支持企业开展高技术、高附加值的保税服务业务。深化自贸区与各口岸通关协作，促进自贸区、综保区等联动发展。高标准建设中法武汉生态示范城，实施中法合作项目三年行动计划，突出国际园区特色，推动绿色高端产业加快集聚。加快建设国家跨境电商综合试验区，深化汉口北国家市场采购贸易方式试点，跨境电商进出口总额增长80%以上。进一步拓展“一带一路”沿线市场，支持行业龙头企业开展国际工程总承包，扩大武汉产品、装备、技术、标准和服务出口。

提升国际通达能力。强化阳逻国际港航运服务功能，加快建设航运产业总部区，建成多式联运海关监管中心，拓展江海直达航线，开行近洋直航航线。强化天河国际机场枢纽功能，新开通国际及地区航线3条以上，全面推进外国人144小时过境免签政策实施，提高航空口岸服务效率。提升中欧（武汉）国际班列平台运营能力，支持中小企业依托班列开展进出口业务。

拓展国际交流合作。实施国际化水平提升计划。推动更多国家在汉设立领事和经贸促进等机构。开展“万名友城市民游武汉”主题活动。全力办好中国2019世界集邮展览，举办世界大健康博览会、中国国际商用车展、武汉国际时装周等国际展会，举办武汉网球公开赛、武汉马拉松、世界飞行者大会、国际篮联篮球世界杯等高规格文体赛事，加快完善国博中心等会展配套设施，打造国际会展之都。

促进区域协调发展。主动服务国家战略，积极发挥在长江中游城市群发展中的引领作用，深化四省会城市会商合作，谋划推进基础设施互联互通和创新资源对接共享。启动大都市区基础设施规划建设工作，加快推动武汉城市圈发展，支持三大国家级开发区与省内市州共建产业园区。优化全市生产力布局，实施区级经济倍增行动，促进各区协调发展。积极申报国家级武汉长江新区，推动长江新城起步区建设，高标准规划建设长江科学城。

武汉，是一座具有变革和开放精神的城市。改革开放40年来，我们创造了一个又一个第一，演绎了“对内搞活看汉正街”的传奇。踏上新征程，我们将坚定改革开放再出发的信心和决心，不断将改革开放推向深入，争做新时代改革开放排头兵！

（五）以筹办军运会为契机，塑造精致武汉新形象

第七届世界军人运动会是党的十九大后我国举办的第一次大型国际体育赛事，也是继北京奥运会后我国举办的规模最大的国际体育盛会。我们将坚持“办好一次会，搞活一座城”，把“办赛事”与“建城市”结合起来，对照“四个一流”，扎实做好筹办和服务保障工作，推动城市面貌脱胎换骨、功能品质系统提升，努力展现一个更干净、更清爽、更有序的大武汉。

做好军运会综合服务保障。严把质量安全关，按时完成场馆设施建设，加快完善场馆周边配套功能。加强公共领域安全监管，做好安保、稳定等工作，提高风险防控和应急处置能力。按照国际赛会要求，全面提升接待、住宿、餐饮、通信、交通、医疗等综合服务水平。引导市民践行东道主文明公约，提高窗口单位服务水平，深入开展“清洁家园迎军运”行动，加强军运会宣传推广，营造热情友好的军运氛围。

推进基础设施提质升级。深入开展综合交通体系三年攻坚行动。实施天河国际机场扩容工程，完成第一跑道大修、T1航站楼改扩建，启动第三、第四跑道建设。推动武（汉）西（安）高铁武汉直通线、新汉阳火车站开工建设，建成光谷火车站，推进高铁进机场。全面提升地铁建设运营质量和水平，加快建设地铁城市，确保开通2号线南延线、8号线三期、蔡甸线，开工建设新港线、12号线三期、11号线葛店段，同步推进10条线路续建，完成新一轮城市轨道交通线网规划修编，启动编制轨道交通第五期建设规划。建成10个公交场站，加强公交线网与地铁站点无缝衔接，努力实现“地铁送到站、公交送到家”。建成杨泗港长江大桥、青山长江大桥、汉江大道、黄家湖大道、光谷广场综合体等项目，开工建设东湖-南湖“两湖隧道”，力争开工建设双柳长江大桥，谋划建设光谷长江大桥。打通10条“断头路”，建成80条微循环道路，大力整治修复提升城市道路。提升市政保障能力，加大停车场、充电桩、加氢站建设力度，新增停车位20万个、充电桩10万个。积极推进骨干排水工程，全市新增抽排能力90立方米/秒。优化供水布局，加快形成全市供水一张网，大力实施二次供水改造工程，改造300处老旧小区二次供水设施，3年内完成全市改造任务。持续推进综合管廊建设。完成海绵城市建设40平方公里。

提升城市精细化管理水平。启用城市总体规划“过渡版总图”，编制完成市级国土空间规划，高标准做好城市设计，加强城市天际线、建筑风格等统筹。聚焦“五边五化”，持续推进综合环境整治提升，以段成线、以块成片，加快建设长江主轴，打造沿江卓越片区，加快推进城市阳台建设；深入开展“城市双修”，继续抓好“三旧”改造，打好老旧小区管网改造、城中村环境整治歼灭战，实施“微改造”，促进城市有机更新。持续推进城管精细化提标行动，全面制订城市管理各领域技术导则，升级新型城市网格化管理体系，推行路长、楼道长、店长“三长制”，进一步下沉执法力量，健全综合执法与行业管理有机衔接机制，加强大数据应用，提高智慧城管水平。大力推进生活垃圾分类工作。继续推进“厕所革命”。

发挥军运会溢出带动效应。实施城市形象海外推广工程，强化城市全球营销，提高武汉国际知名度。加大武汉文化旅游品牌境外推介力度，打造全球旅游目的地城市。开展主题消费活动，扩大消费需求。加强军运会场馆设施、文化品牌等综合开发和深度利用。

（六）持续改善生态环境，提升城市宜居品质

强力推进共抓长江大保护。继续打好长江大保护十大标志性战役，落实长江经济带绿色发展十大战略性举措，建设国家长江经济带绿色发展示范区。大力推进武汉长江汉江核心区港口码头岸线资源优化调整，严厉打击非法采砂，实现“一无一化一提升”。深化“一城一港一主体”，提高港口运营管理效率，加快推进阳逻国际港铁水联运二期项目，完善港口集疏运体系，开展港口绿色化改造。持续做好沿江化工企业关、改、搬、转工作。

实施全域绿化提升行动。积极创建国家生态园林城市，加大长江主轴、东湖生态绿心、府河生态湿地、天兴洲生态绿洲等重点区域生态保护和建设力度，加快推进长江两岸造林绿化、四环线生态带等重大绿化项目建设，新建各类公园45个，新增绿地600万平方米，新建绿道300公里，植树造林2万亩。精心做好园林绿化日常管护。加强矿山修复整治工作。巩固精准灭荒成果。

健全生态环境治理体系。深化完善长江汉江武汉段跨区断面水质考核奖惩及生态补偿制度，严格落实河湖长制。加快建设全国碳交易注册登记系统，推进碳排放权和主要污染物排污权交易。落实生态环境损害赔偿制度改革。加强领导干部自然资源资产离任审计。推动修订机动车排气污染防治条例。健全跨部门、跨区域环保应急协调联动机制。开展生态环保全民行动。

（七）深入实施乡村振兴战略，促进城乡融合发展

大力发展现代都市农业。创新提升“三乡工程”，持续推动人才、资本、技术下乡，打造“一乡一村一品”。大力推进都市田园综合体建设，推动农村产业融合发展。加快建设“中国种都”。实施农产品加工业提升行动，进一步提高农产品质量安全水平，农产品加工业实现产值2900亿元。大力发展农村电商，农产品网络零售额突破250亿元。推动农民就地就近就业创业。

深化农村改革。完善农村土地所有权、承包权、经营权“三权分置”制度，探索宅基地所有权、资格权、使用权“三权分置”改革办法，进一步盘活农村土地资源。全面推进农村集体产权制度改革。创新农业经营方式，实施新型农业经营主体培育工程，大力培育新型职业农民，新增家庭农场100家，新培育农民专业合作社310家，培育认证安全优质农产品品牌。

深入推进美丽乡村建设。健全现代乡村治理体系，提升乡村自治法治德治水平，培育良好文明乡风，建设平安乡村。全面开展村庄规划编制，深入推进“四个三重大生态工程”，加强村湾环境整治，完成33座农村老旧简易垃圾填埋场生态修复，实现农村地区无敞口垃圾池；完成765座农村公共厕所建设改造、12万户农村户厕改造任务；开展1000个行政村生活污水治理；对300公里通客运班车的农村公路提档升级，新建通湾公路200公里；创建省级绿色乡村45个。

（八）提高保障和改善民生水平，让市民共享改革发展成果

促进更高质量更充分就业。实施就业优先战略和积极就业政策，扶持创业3万人，城镇新增就业20万人。支持建设大学生就业见习基地20个以上、实习实训基地200个以上。加强城镇就业困难人员就业援助，动态消除“零就业家庭”。优化创业贷款担保服务，将农村自主创业农民纳入支持范围。继续为武汉户籍外出务工农民购买人身意外伤害保险。大规模开展职业技能培训。申报创建国家级人力资源服务产业园。开展失业保险援企稳岗“护航行动”，扩大稳岗补贴覆盖范围。深入实施城乡居民增收激励计划。继续提高退休人员基本养老金。做好农民工工资清欠保支工作。完善政府、工会、企业共同参与的协商协调机制，构建和谐劳动关系。

织牢社会保障网。推进全民参保，社保扩面新增40万人次。提高城乡低保、抚恤优待、基本医疗保险财政补助等标准。为全市贫困妇女购买特定医疗安康保险。落实残疾儿童康复救助制度。大力发展公益慈善事业。深入推进全国养老服务业综合改革试点，推动城市适老化改造，新建社区老年人服务中心（站）100家、农村老年人互助照料活动中心（服务点）307个，开展长期护理保险制度试点。加快棚户区改造，新开工3.5万套，建成2.5万套，建设筹集公租房1万套、大学毕业生保障房40万平方米。

发展公平而有质量的教育。深化教育领域综合改革。新改扩建公益普惠性幼儿园30所，新增学位8000个，公办幼儿园和普惠性民办幼儿园在园幼儿占比达到80%以上。新改扩建公办中小学20所，新增学位2.4万个。提升43所乡村小规模学校和104所乡镇寄宿制学校办学条件。大力改善农村公办幼儿园办园条件，建设农村留守儿童关爱服务站10家、青少年寒暑假社区托管室240家。综合防控儿童青少年近视。办好特殊教育，提高职业教育发展水平。加强校园安全管理。

提高公共文化服务质量。积极践行社会主义核心价值观，推进“红色基因”传承工程，深入实施公民道德建设工程，深化文明城市建设。打造“长江文明之心”，推动建设世界级历史人文集聚展示区。规划建设新长江文明馆，建成开放盘龙城国家考古遗址公园、琴台美术馆。建设武汉戏曲艺术中心。推进万里茶道申报世界文化遗产。深化国家文化消费试点。开展高雅艺术惠民演出季活动，举办琴台音乐节、武汉高校艺术节等活动。组织“武汉之夏”等文化惠民活动3000场次，开展“家庭教育公益大讲堂”进社区（村）2000场。推进国家足球发展改革试点。举办第七届全民健身运动会，更新和新建社区（村）健身器材500套。大力发展全域旅游，推进文化旅游深度融合，积极整合提升“两江四岸”旅游资源，规划建设一批旅游特色街区和景区，打造一批经典文化旅游线路。激发文化创新创造活力，加快培育数字创意、设计服务等新型文化业态，扩大“设计之都”品牌效应，支持各区打造特色文化创意产业园区。

提升居民健康水平。推进健康武汉建设。深化公立医院综合改革，扩展药品带量采购范围和品种，进一步控制医疗费用不合理增长。完善分级诊疗制度，巩固医疗联合体建设。深化基层医疗卫生综合改革，开展“优质服务基层行”，促进4家基层医疗机构提档升级，5个区级疾控中心达到国家标准。加强公共卫生体系建设，推进区域医疗中心建设，加快建设市儿童医院妇幼综合大楼等项目，建成投用市急救中心、市疾控中心等迁建项目。传承发展中医药事业。培育发展大健康产业。巩固国家卫生城市创建成果。推动无烟环境建设和控烟立法。提升国家食品安全示范城市建设水平，完善全链条可追溯监管体系，健全药品监管长效机制，坚决维护人民群众身体健康。

深化社会治理创新。健全城乡社区治理体系，推广百步亭、青和居等社区治理经验，扩大老旧小区“红色物业”覆盖面，加大对特殊群体的监护、关爱力度。加强城乡社区综合服务设施建设，建成社区社会工作服务站100家，推进100个农村社区建设试点，探索在有条件的农村社区开展物业服务。纵深推进“万名警察进社区”活动，建设500个“智慧平安小区”，星级平安社区（村）覆盖率达50%，健全社会治安防控体系。深入推进扫黑除恶专项斗争。深化信访工作制度改革和信访法治化建设，积极化解信访积案，着力解决群众反映突出的历史遗留问题。依法加强互联网综合治理。深入实施“七五”普法，完善公共法律服务体系。继续做好双拥共建、国防动员工作，逐步健全完善退役军人服务保障体系。开展安全生产隐患排查与治理，加强综合性消防救援力量建设，做好事故管控工作，确保安全生产形势总体平稳。

各位代表，为民的事没有小事。一段时期以来，我们每年提出为民办理十件实事，收到积极成效。考虑到十件实事已经涵盖不了政府要办的所有实事，从今年开始，我们在《政府工作报告》中不再单列十件实事，把政府当年要办的实事，分列到政府各部门工作中，强化常态化办理机制，进一步加大办理力度，努力把实事办的更多、办的更好、办的让市民更满意！

三、建设人民满意的服务型政府

民心是最大的政治，人民满意是我们的最高追求。我们要对照“五个过硬”，进一步转变政府职能，以新时代新担当新作为，高水平做好政府各项工作。

坚决做到“两个维护”。以政治建设为统领，树牢“四个意识”，坚定“四个自信”，坚决维护习近平总书记核心地位，坚决维护党中央权威和集中统一领导，坚决贯彻党中央决策部署，坚决落实省委省政府和市委部署要求。全面落实意识形态工作责任制。认真抓好中央和省委巡视反馈意见整改落实。

坚持依法行政。全面落实法治政府建设实施方案。加强重大行政决策、规范性文件、重大行政执法决定合法性审查，提高政府决策科学化、民主化、法治化水平。推动政府机构改革，深化综合行政执法改革。优化政府办事规程，加大政务公开力度。深入推进行政机关负责人出庭应诉，认真执行法院生效裁判。严格执行市人大及其常委会的决议决定，自觉接受人大监督、政协监督，自觉接受监察监督，加强审计监督，主动接受社会和舆论监督，确保行政权力在法治轨道上运行。

强化担当作为。全面增强“八个本领”，履职尽责、敢抓敢管，创造性开展工作，勇当改革发展“主攻手”。建立健全激励约束机制和容错机制，保护和调动广大干部干事创业积极性。深入开展“大调研大落实”，驰而不息纠正“四风”，力戒形式主义、官僚主义，把更多时间用在抓工作落实上。

加强廉政建设。切实履行全面从严治党主体责任和“一岗双责”，深入推进政府系统党风廉政建设和反腐败工作。严格执行廉洁自律准则和纪律处分条例，严格落实中央八项规定实施细则以及省、市实施办法。加强公共资金、国有资产、国有资源等领域廉政风险防控，从源头预防腐败滋生。

各位代表！新的一年充满机遇，也面临挑战，让我们更加紧密地团结在以习近平同志为核心的党中央周围，在省委省政府和市委的坚强领导下，以高昂的奋进姿态，乘势而上、迎难而上，奋力谱写“三化”大武汉和国家中心城市建设新篇章，以优异成绩向新中国成立70周年献礼！
